# Herbal triplet in treatment of nervous agitation in children

**DOI:** 10.1007/s10354-012-0165-1

**Published:** 2012-11-22

**Authors:** Inga Trompetter, Bianka Krick, Gabriele Weiss

**Affiliations:** PASCOE pharmazeutische Präparate GmbH, Schiffenberger Weg 55, 35394 Giessen, Germany

**Keywords:** Nervous agitation, Anxiety, Depression, Plant extracts, Children, Nervöse Unruhe, Angst, Depression, Pflanzenextrakte, Kinder

## Abstract

Emotional and behavioral problems in children and adolescents are no exception. To what extent a fixed plant extract combination is able to support children suffering from nervous agitation due to agitated depression among others for approximately 2 years has been investigated in a multicenter, prospective observational study (2008) with 115 children between 6 and 12 years. Assessments of the parents showed a distinct improvement in children who had attention problems, showed social withdrawal, and/or were anxious/depressive. Based on the physicians’ assessment, 81.6–93.9 % of the affected children had no or just mild symptoms at the end of observation concerning nine of thirteen evaluated symptoms such as depression, school/examination anxieties, further anxieties, sleeping problems, and different physical problems. Therapeutic success was not influenced by additional medication or therapies. The treatment was well tolerated. The used plant extracts have been gained from St. John’s Wort herb, valerian root, and passionflower herb.

## Abstract (de)

Emotionale Probleme und Verhaltensauffälligkeiten bei Kindern und Jugendlichen sind keine Ausnahme. Inwieweit Kindern, die seit etwa 2 Jahren unter nervöser Unruhe (unter anderem aufgrund agitierter Depression) litten, durch eine Behandlung mit einem pflanzlichen Kombinationspräparat geholfen werden kann, zeigt eine multizentrische, prospektive Beobachtungsstudie (2008) mit 115 Kindern im Alter von 6 bis 12 Jahren. Nach Einschätzung der Eltern zeigte sich eine klare Besserung insbesondere bei Konzentrationsstörungen, sozialem Rückzug, bei Kindern, die unter Ängsten und/oder Depressionen litten. Basierend auf der Beurteilung der Ärzte waren 81,6–93,9 % der Kinder am Ende der Beobachtungszeit beschwerdefrei oder hatten nur noch leichte Beschwerden in Bezug auf neun der dreizehn untersuchten Symptome, wie Depressionen, Schul-/Prüfungsangst, sonstige Ängste, Schlafstörungen und verschiedene physische Probleme. Der therapeutische Erfolg war unabhängig von weiteren Therapien oder Begleitmedikation aufgrund der Einschlussdiagnose. Die Behandlung wurde gut vertragen. Die verwendeten Pflanzenextrakte wurden aus Johanniskraut, Baldrianwurzel und Passionsblumenkraut gewonnen.

## Introduction

Especially, young people need a strong mental well-being to cope with all the intricacies of life. However, during the last 16 years, the number of children and adolescents showing psychosomatic symptoms is consistently high. There might be no epidemic [1], but the following numbers are alarming anyway. In any given year, worldwide 20 % of young people experience a mental health problem, most commonly depression or anxiety [2]. In Austria, pupils every day or at least several times a week suffer from sleep disorders (17.2 %), headache (14.4 %), petulance (14.2 %), nervousness (11.7 %), and/or backache (11.7 %) [3]. Similar numbers have been measured in other countries, e.g., in Canada, 21–27 % of grade 6 adolescents feel depressed at least once a week, 21 % of children at 2–5 years of age suffer from high levels of emotional problems, anxiety problems, lack of concentration, and/or hyperactivity [4, 5].These numbers are similar in other countries. In Germany, every sixth child or adolescent shows psychiatric disorders [6, 7].

There is a scientific consensus that these children (and their families) have to be supported and treated with adequate therapies [7–9], at least because children with anxieties and depressive episodes are at increased risk for severe emotional problems in adolescence and adulthood [10]. The broad range of interventions for anxieties, nervousness, and depression involve cognitive behavioral therapy as well as mostly chemical antidepressants, anxiolytics, and hypnotics [11–13]. Especially, treatment with traditional allopathic medication is controversially discussed addressing efficacy and safety of psychotropic agents in pediatrics. Due to the potential for side effects and addiction, prolonged treatment with chemical drugs as it is recommended by the WHO is often accompanied by simultaneous impairment of quality of life [7, 9, 12, 14, 15]. As a result, there is a clear increasing demand for complementary and alternative medicine [16] such as a unique combination of extracts from *Hypericum perforatum* (St. John’s Wort), *Passiflora incarnata* (Passionflower), and *Valeriana officinalis* (Valerian). Beyond traditional knowledge about the usage of *Hypericum* in treatment of mood and sleep disorders, recent studies have shown various effects on central neurotransmitter systems [17, 18] and the ability to act similar to conventional antidepressive drugs [18–20]. The experimentally verified synergistic effect of *Passiflora* on *Hypericum* enables application of a small amount of *Hypericum* with a simultaneous high efficacy. This reduces the probability of side effects [21] and leads to effects more comparable to the impact of Fluoxetine than to the impact of St. John’s Wort extract alone [22]. *Passiflora* is traditionally used in combination with other herbs as a mild sedative [23, 24]. The third plant, Valerian, is traditionally used in medical conditions of sleep disorders and nervous agitation [25]. The combination of all the three medical plants act on gamma-amino butyric acid (GABA) and serotonin (5-HT) receptors, which are recognized targets of pharmacological antidepressant treatment [26].

The aim of this observational study was the evaluation of safety and effectiveness of the treatment of nervous agitation due to affective disorders in children between 6 and 12 years of age with a fixed herbal combination containing St. John’s Wort, Valerian, and Passionflower.

## Material and methods

### Study objectives

After more than 40 years of experience with these plant extracts, we hypothesized that a combination of St. John’s Wort, Valerian, and Passionflower has positive impact on children with nervous agitation due to affective disorders.

### Study duration

The duration of the study was from March 2008 to November 2009.

### Study design

This multicenter, prospective observational study was conducted by 17 physicians (15 pediatricians, 1 neurologist, and 1 general practitioner) in Germany.

Concept and design of the study as well as its ethical validity and performance are based on the actual recommendations of the Bundesinstitut für Arzneimittel und Medizinprodukte (BfArM, German Federal Institute for Drugs and Medical Devices) and referred to the Declaration of Helsinki and Good Clinical Practice. The study was registered on Clinical Trials under the trial registration number NCT 01125579. Furthermore, this study followed the actual strengthening the reporting of observational studies in epidemiology (STROBE) guidelines for items to be included in reports on observational studies [27].

### Study setting

Before the start of this study, the physicians got a briefing on the observational plan, the ethical and scientific basis of this multicenter, prospective observational study with observational character, the allocation procedure, and the therapy schedule in accordance with the study protocol. Data assessment included a physician-completed questionnaire and a standardized parent-report questionnaire (Child Behavior Checklist (CBCL/4-18)) on three occasions: before the treatment (visit 1, baseline), after approximately 2 weeks of the treatment (visit 2), and after approximately 4 weeks of the treatment (visit 3). The time point of final documentation was set at the physicians’ own discretion. The concomitant diseases were grouped according to the ICD-10 classification. All medications apart from the study medication were classified according to the—to that time current—“Rote Liste 2008”.

### Study participants

The children were chosen by the responsible physician and enrolled after the informed written consent, and a data privacy policy statement had been obtained from their legal guardian. Information of possible unwanted side effects and the possibility to cancel the participation at any time without any negative impact on the treatment of the child were given to the legal guardian.

Inclusion criteria were defined as follows: 6–12 years of age, history of nervousness and agitation (including agitated depression) due to affective disorders, informed consent to participate in the prospective cohort study obtained from the legal guardian. Exclusion criteria contained children younger than 6 years or older than 12 years of age, hypersensitivity to any of the ingredients, history of skin hypersensitivity to light, receiving phototherapy, or any photodiagnostic procedures.

### Variables studied

Target parameters for effectiveness were the influence of child’s disease on everyday life of the family, the course of common symptoms, and the change in the parent’s questionnaire CBCL (Table 1).

**Table 1:** Study course

Assessment criteria	Visit 1	Visit 2	Visit 3
Demographic data (sex, age, body height, and body weight)	X	–	–
Concomitant therapy due to inclusion diagnosis	X	–	–
Concomitant medication in general and due to inclusion diagnosis	X	–	–
Efficacy and tolerability of previous therapy due to inclusion diagnosis	X	–	–
Impairment to everyday life of the family by child’s complaints (filled in by legal guardian; visual analogue scale; 0 = no limitation; 10 = extreme limitation)	X	X	X
CBCL/4-18 (filled in by legal guardian)	X	X	X
Common symptoms such as learning disorders, speech disorders, lack of concentration, school/examination anxieties, other anxiety disorders, aggressiveness/irritability, depression, uncoordinated hyperkinesia, tiredness/fatigue, problems falling asleep, problems staying asleep, headache, and abdominal/stomach pain (filled in by physician; 0 = nothing; 3 = strong)	X	X	X
Drug changes (newly prescribed, withdrawn, dosage decreased, and dosage increased)		X	X
Tolerability	–	X	X
Adverse drug reactions	–	X	X

*CBCL* child behavior checklist

### Interventions

Doses and duration of the treatment with herbal medicine were at the respective physician’s discretion. The combination of three special dry extracts from *Hypericum perforatum* (St. John’s Wort herb), *Valeriana officinalis* (Valerian root), and *Passiflora incarnata* (Passionflower herb; Table 2) has been administrated as tablet via oral route. Study medication was prescribed by a physician and bought by the patient’s legal guardian.

**Table 2:** Composition of one tablet of study medication

Active ingredient	Extractant (m/m)	Drug–extract ratio	Milligram/tablet
Dried extract of St. John’s Wort herb (*Hypericum perforatum* L.)	Ethanol 38 %	4.6–6.5:1	60
Dried extract of Valerian root (*Valeriana officinalis* L.)	Ethanol 40 %	3.8–5.6:1	28
Dried extract of Passionflower herb (*Passiflora incarnata* L.)	Ethanol 60 %	6.25–7.1:1	32

## Results

### Participants

Altogether 31 physicians were invited to collect data and 17 physicians agreed to participate in the study. They gathered correctly and completely filled-out case report forms of 115 children. Therefore, the participant rate was 54.8 %.

### Descriptive data

The patient group involved in this study consisted of 69 boys (60 %) and 45 girls (39.1 %). No statement of sex was made for one child, which explains the missing 0.9 %. In average, the children were 9.4 years old (± 1.6) with an average weight about 35.4 kg (± 9.6). The inclusion diagnosis “nervous agitation” was made on average about 2.3 years ago. So, in the huge majority of the treated children (92.6 %), a subchronic or chronic pattern of symptoms was already present.

Period of the treatment enfolded 2 weeks (*n* = 14), including visit 1 and visit 2 or about 4 weeks (*n* = 101) including all the three visits (Table 1). The average daily dose was 2–3 tablets in accordance with the recommend dose of 1–3 tablets per day.

During the observation period, 16 children (13.9 %) received additional medications related to the inclusion diagnosis, mainly hypnotics/sedatives (64 %) and psychopharmaceuticals/psychoanaleptics (12 %). Eighty-four percentages of the respective drugs were used as long-term medication. Up to three nonmedical therapies were reported for 61 children (53 %) such as psychological/psychotherapeutic care (46.6 %) and progressive relaxation (19.2 %).

Concomitant diseases were mentioned for 21 children (18.3 %), mainly emotional and behavioral disorders (40.7 %) and diseases of the respiratory system (18.5 %). For 14 children (12.2 %) concomitant medication was reported, mainly broncholytics/antiasthmatics, antiallergics, and dermatologics.

### Impairment of ordinary family life

Behavioral disorders of children and their therapies always affect the everyday family life. At the first visit, parents estimated the impact via a visual analogue scale with 5.95 in the mean. After the treatment, this average value improved to 3.86. Altogether, a clearly positive development was achieved in 86.7 % of children.

### Child behavior questionnaire

Evaluation of the CBCL displays a detailed picture of the parents’ assessment from the beginning until the end of the observation time. In comparison to children of the same age, most of the children (up to 87.0 %) were assessed as “normal” concerning items of the competency scales at all visits. Some children showed aspects of “borderline abnormal” (up to 11.3 %) or “abnormal” (up to 13.0 %) behavior.

Until the end of the observation most children kept their level. According to which the competency scale is viewed two to five children aggravated in their problems, while an improvement was achieved for nine to ten children.

**Fig. 1:**
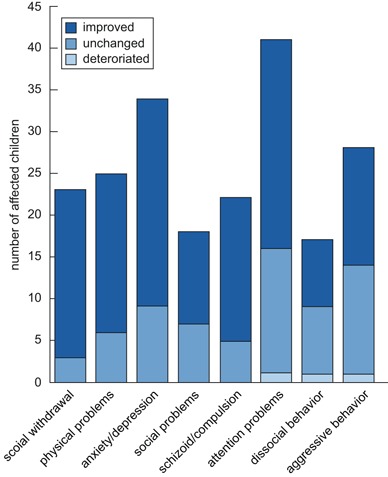
Change in syndrome scales until the end of the observation period. Depiction of the number of affected children, whose problems improved, remained unchanged, or deteriorated during the treatment. The classification in syndrome scales depended on the Child Behavior Checklist

Analysis of syndrome scale items also showed that most children behaved “normal” (85.2–64.3 %, depending on the scale). The majority of children were stabilized in a normal behavior under the treatment. Slight aggravation of single participants was detectable in items of a schizoid/compulsive (four children) or dissocial behavior (three children). The highest proportion of “borderline normal” or “abnormal”-behaving children were detectable in the two scales “attention problems” (35.7 %) and “anxiety/depression” (29.6 %) (Fig. 1).

Figure 1 gives an overview of the number of affected children, and the development they underwent during the therapy. A clear improvement was reported by legal guardians, whose children had attention problems, showed social withdrawal, and/or were anxious/depressive. Between 61.0 and 87.0 % of the affected children developed positively. In the same range was the progress of children with physical and/or social problems. Even children with a schizoid/compulsive behavior improved in their development. A little lower was the percentage of children with a clear progress when the participants showed dissocial (47.1 %) and/or aggressive behavior (50 %) at the beginning of the observation.

Altogether, up to 69.6 % of children who were assessed as “borderline abnormal” or “abnormal” reached a “normal” assessment at the end of the observation period. The therapeutic success was not influenced by concomitant medication or therapies due to inclusion diagnosis.

### Assessment of common symptoms by the physician

**Fig. 2:**
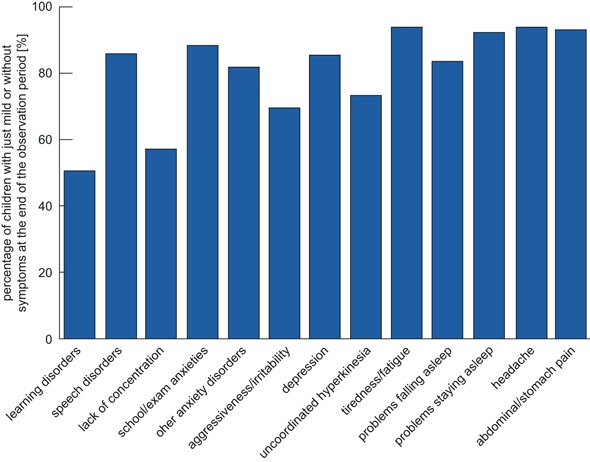
Affected children with just mild or without symptoms at the end of the observation period. Classification of children based on the assessment of the attending physician

The statements about the problems of children with nervous agitation given by the CBCL were confirmed by physicians in aspects such as the high number of children affected by attention problems, anxiety/depression, aggressiveness, and/or physical problems. In addition to this, the results of the symptom query presented more details in some aspects. The scale “anxiety/depression” is divided into three symptoms, which showed that the amount of children suffering from examination anxieties (59.1 %) or other anxiety disorders (42.6 %) was much higher than the number of depressive children (29.6 %) in this study. Physical problems are also mentioned for lots of children such as tiredness/fatigue (54.8 %), abdominal/stomach pain (39.1 %), and/or headache (42.6 %). Problems to fall or stay asleep were mentioned in 53.9 and 33.9 % of the children, respectively. After the treatment, 81.6–93.9 % of the affected children had no or just mild symptoms concerning nine of thirteen evaluated symptoms such as depression, school/examination anxieties, further anxieties, sleeping problems, and different physical problems (Fig. 2). The percentage of children suffering from symptoms such as uncoordinated hyperkinesia, aggressiveness/irritability, lack of concentration, and learning disorders was clearly reduced in the end, too. Between 50.8 and 73.0 % of children with these problems had no or just mild symptoms in the end.

In general, concomitant therapies or medication due to inclusion diagnosis did not affect the efficacy. At the end of the observation period, the number of children with just slight or no problems tended to be higher in cases without concomitant medication in learning problems/partial performance problems and problems in falling asleep. A tendency for a better result with concomitant medication was reported for compulsive uncoordinated movements.

### Tolerability

A good tolerability was reported for 97.4 % of the children. In seven cases, the tolerability was described as moderate or poor, because of paradox reactions (restlessness, weepiness, increased irritability, or aggressiveness) or other adverse events such as redness in cheekbone region (one case) or stomach pain (one case). All events were considered to have a possible relation to the study treatment.

In 53 children (46.1 %), the therapy was continued after the end of the prospective cohort study.

## Discussion

Contrary to various other diseases, nervous agitation due to depressive episodes is not measurable by definite laboratory findings. Each child shows an individual set of few to various symptoms with further variations in intensity. This is also reflected by the assessments of parents, other legal guardians, and physicians taking part in the presented study. Typical symptoms of the examined children were attention problems, anxieties, depression, and psychosomatic problems. However, approximately 40–50 % of children with these disorders do not have a response to medication or behavioral therapy alone [28, 29], and a combination of both is more effective [30]. On the other hand, psychotropic medications are used too early [7] and about 25 % of depressive adolescents develop substance abuse [31]. So, parents prefer nonmedical therapies as initial treatment because of the higher risk of side effects in therapies with chemical medication [30]. For these families, the tested herbal combination of St. John’s Wort, Valerian, and Passionflower offers a good alternative and fulfills the requested aspects. A good tolerability is represented by just a few mild and transient side-effects. The assessments of parents and physicians displayed a good efficacy on a broad spectrum of symptoms linked to inclusion diagnosis. Moreover, it is possible to adjust the treatment individually because the good efficacy was independent from concomitant nonmedical and medical therapies—with three exceptions. In situations of learning problems and problems falling asleep, the treatment without concomitant drug therapy due to inclusion diagnosis tended to be more successful. In comparison to that treatment of uncoordinated hyperkinesia seemed to be more effective with concomitant medication.

Another advantage for use of the study medication in initial therapies is the fast onset: 3 h after first intake [22]. The experiences collected by long marketing history and supported by the recent results of the presented study showed that the study medication is useful in situations of psychological mood disorders and sleep disorders due to nervousness also in children between 6 and 12 years of age.

## Limitations

Due to the officially required design of a multicenter, prospective observational study some limitations are given. Therefore, it is unavoidable that safety and effectiveness are observed without a placebo group and randomization. Lack of blinding, the subjectivity of assessments, and the potential impact of the relationship between child, parents, and physician on the child’s development may also be limitations. On the other hand, this type of study offered the possibility to prove the medication’s tolerability and effectiveness in everyday life beyond a strictly controlled environment of a clinical study.

### Acknowledgments

We thank all physicians, parents, and children who participated in the study.
